# High-Stability Lithium Metal Batteries Enabled by AZO-Modified Separators

**DOI:** 10.3390/ma19071429

**Published:** 2026-04-03

**Authors:** Shaojiang Hong, Ruiqin Tan, Jia Li, Jinhua Huang, Weijie Song

**Affiliations:** 1 Faculty of Electrical Engineering and Computer Science, Ningbo University, Ningbo 315211, China; 2 Ningbo Institute of Materials Technology and Engineering, Chinese Academy of Sciences, Ningbo 315201, China

**Keywords:** lithium metal batteries, lithium dendrites, separator modification, AZO, magnetron sputtering

## Abstract

The commercialization of lithium metal batteries is hindered by critical challenges such as uncontrollable lithium dendrite growth and interfacial instability. Constructing functional nanocoatings on separator surfaces represents an effective strategy to address these issues. In this study, a uniform aluminum-doped zinc oxide (AZO) modification layer was deposited on the separator via magnetron sputtering to enhance the electrochemical performance and safety of lithium metal batteries. The AZO layer combines the functions of a physical barrier and an interfacial regulator. On one hand, it effectively suppresses lithium dendrite penetration through the separator. On the other hand, its surface properties facilitate uniform lithium-ion transport and reduce the deposition overpotential. Experimental results demonstrate that the symmetric cells employing AZO-modified separators exhibit significantly reduced and stable lithium deposition overpotentials. In full cells assembled with a nickel cobalt aluminum (NCA) cathode, the system demonstrates higher specific capacity and notably extended cycle life compared to cells using unmodified polyethylene (PE) separators. This work proposes a practical strategy based on AZO-modified separators, offering a promising pathway toward the development of next-generation lithium metal batteries with high energy density and improved safety.

## 1. Introduction

In the 1970s and 1980s, the use of lithium metal anodes in secondary batteries was largely abandoned due to safety concerns—a setback that severely hindered their commercial development. It is worth noting that alongside the development of lithium-based systems, aqueous rechargeable batteries have also been demonstrated and successfully commercialized for specific applications, offering inherent safety advantages and lower cost [1,2]. However, the recent rapid expansion of electric vehicles and wearable devices has created a compelling demand for batteries with higher energy density [3,4,5,6,7]. With its ultra-high theoretical specific capacity of 3860 mAh g^−1^, lithium metal has re-emerged as a promising candidate [8,9,10].

Despite renewed interest, the commercialization of lithium metal batteries still confronts several critical challenges. First, the high reactivity of lithium metal induces continuous parasitic reactions with the electrolyte, forming a solid electrolyte interphase (SEI) layer [11,12,13]. This SEI is often unstable and susceptible to fracture [14,15] leading to sustained lithium corrosion and electrolyte depletion. Second, lithium dendrites that form during cycling can penetrate the separator, causing internal short circuits [16]. As these dendrites become denser and elongated, they further exacerbate electrolyte consumption. When deposited lithium loses electrical contact with the anode, it becomes electrochemically inactive, resulting in the formation of “dead lithium” [17], which causes irreversible capacity loss [18,19,20]. To mitigate these issues, various strategies have been pursued, including the use of electrolyte additives [21,22], the design of functional current collectors [23,24], anode interface engineering, and separator modification [25,26].

To date, extensive research has focused on inorganic oxide coatings—such as ZnO, MgO, and Al_2_O_3_—applied to various battery components to enhance electrochemical stability [27,28,29]. Among these, aluminum-doped zinc oxide (AZO) has attracted particular attention due to its unique combination of properties. Bai et al. [30] demonstrated that AZO coating on electrolytes effectively suppresses lithium dendrite growth and improves cycling performance in lithium metal batteries, while Dai et al. [31] reported that direct AZO coating on lithium cobalt oxide (LiCoO_2_) electrodes provides more efficient electron transport and a stable interfacial layer. These studies establish AZO as a promising material for interfacial modification. However, despite its demonstrated potential in other battery components, systematic investigations of AZO-coated separators for lithium metal batteries remain scarce in the literature.

The selection of AZO among various inorganic oxides is motivated by several key considerations. While pure ZnO—a wide-bandgap semiconductor—has been explored for battery applications, its intrinsic resistivity (>10^6^ Ω·cm) limits its effectiveness in regulating interfacial charge distribution [32]. Aluminum doping addresses this limitation: Al^3+^ substitution of Zn^2+^ sites generates free electrons, reducing resistivity to the 10^−4^ Ω·cm level and yielding moderate electrical conductivity (typically 10^−3^–10^2^ S·cm^−1^) [33]. This conductivity level enables AZO to participate actively in regulating interfacial charge distribution, distinguishing it from conventional insulating ceramics such as Al_2_O_3_ and MgO (resistivity > 10^14^ Ω·cm), which function purely as passive physical barriers. Compared to highly conductive metallic coatings (e.g., Cu, ∼10^5^ S·cm^−1^) that may promote undesirable electronic leakage [34]. AZO’s intermediate conductivity strikes an optimal balance between sufficient electronic transport for current homogenization and avoiding excessive conductivity that could compromise interfacial stability. Beyond its conductivity, AZO preserves the lithiophilic nature of ZnO, providing favorable nucleation sites for uniform lithium deposition, and can be deposited as uniform thin films via magnetron sputtering—a technique that ensures superior adhesion and coverage uniformity on polyolefin separators. By depositing AZO under optimized conditions, we achieve a coating that synergistically integrates structural robustness, moderate electrical conductivity, chemical stability, and lithiophilic surface chemistry—a combination of properties that remains scarce in separator coating materials [35]. This work provides the first systematic investigation of AZO-coated separators for lithium metal batteries, offering a meaningful contribution in this direction.

In this work, a uniform aluminum-doped zinc oxide (AZO) coating was deposited onto the surface of a battery separator via magnetron sputtering. This technique not only ensures superior adhesion and uniform coverage between the coating and the separator but also confers unique multifunctional properties upon the separator. The resulting AZO-modified separator synergistically integrates structural robustness with moderate electrical conductivity. Experimental results reveal that lithium metal batteries incorporating the AZO-modified separator exhibit markedly reduced polarization voltage, superior cycling stability, and enhanced capacity retention, thereby validating the efficacy of this strategy in suppressing dendrite growth and improving battery stability.

## 2. Materials and Methods

### 2.1. Magnetron Sputtering of AZO Modification Layer

The AZO target (ZnO:Al_2_O_3_ = 98:2 wt%) was obtained from Zhongnuo New Material Technology Co., Ltd. (Beijing, China). A uniform AZO modification layer was deposited onto a commercial PE separator (supplied by Tianjin Annuohe New Energy Technology Co., Ltd. (Tianjin, China)) via direct current (DC) magnetron sputtering at room temperature. Prior to deposition, the chamber was evacuated to a base pressure of 8 × 10^−4^ Pa. Sputtering was conducted at a working pressure of 0.4 Pa under an argon atmosphere with a DC power of 200 W. The target was pre-sputtered for 10 min to remove surface contaminants. The deposition time was optimized to 30 min, yielding a uniform AZO layer with a thickness of 150 nm.

### 2.2. Material Characterization

Scanning Electron Microscope (SEM) images and Energy Dispersive Spectrometer (EDS) spectra were acquired using a Verios G4 UC SEM (Thermo Fisher Scientific, Hillsboro, OR, USA). The thickness of the prepared films was determined by fitting using an M-2000DI ellipsometer (J.A. Woollam Co., Lincoln, NE, USA). An AXIS ULTRA DLD X-ray Photoelectron Spectrometer (XPS) (Kratos Analytical Ltd., Manchester, UK) was used to detect and analyze the chemical states of elements.

### 2.3. Measurement Method of AZO Coating Thickness

First, a spectroscopic ellipsometer (M-2000DI) was used to measure the thin film deposited on a silicon wafer from an AZO target under identical sputtering conditions. The ellipsometric data were fitted using the Cauchy model to obtain the film thickness (50 nm). Based on the fitted film thickness and the magnetron sputtering time (10 min) on the silicon wafer, the sputtering rate of the AZO target under these conditions was calculated to be 5 nm/min. Subsequently, the desired AZO coating thickness can be obtained by controlling the sputtering time under the same sputtering conditions.

### 2.4. Electrochemical Testing

CR2032-type coin cells were assembled and tested in an argon-filled glove box, configured as Li||Li symmetric cells and full cells. For symmetric cell testing, the PE separator was modified on both sides via magnetron sputtering, and the resulting double-side modified separator was employed. For full cell assembly, a single-side modified separator was used, with the modified side facing the lithium metal anode. NCA served as the cathode material. The cathode slurry was prepared by uniformly mixing NCA active material, Super P conductive carbon black, and polyvinylidene fluoride (PVDF) binder at a specific weight ratio in N-methyl-2-pyrrolidone (NMP) solvent. The resulting slurry was then coated onto an aluminum foil current collector and vacuum-dried at 120 °C. The average active material mass of the NCA cathode electrodes was 24.5 mg per electrode. The electrolyte consisted of 1.0 M lithium hexafluorophosphate (LiPF_6_) in a mixture of fluoroethylene carbonate (FEC) and ethyl methyl carbonate (EMC) (1:5 by volume) with 0.8 wt% lithium difluorophosphate (LiPO_2_F_2_) as an additive. Each coin cell received 75 μL of electrolyte. All electrochemical tests were performed using a Land battery testing system.

## 3. Results and Discussion

**Figure 1:**
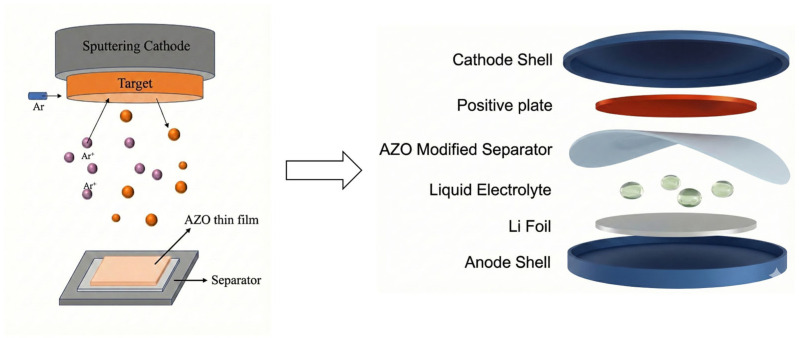
Preparation of AZO-modified separator and assembly of lithium metal coin cell.

The AZO modification layer was deposited onto the separator via magnetron sputtering, and the resulting modified separator was subsequently assembled into coin cells (Figure 1). To balance effective regulation of lithium-ion flux and dendrite suppression against potential drawbacks such as increased internal resistance or mechanical degradation during cycling—both of which may arise from excessive coating thickness—two specific thicknesses, 150 nm and 200 nm, were selected. During magnetron sputtering under an argon plasma, the AZO target was bombarded, leading to the uniform deposition of a dense AZO film on the separator surface. This technique enables precise control over film thickness and composition, ensuring nanoscale uniformity and consistency of the modification layer, thereby establishing a reliable foundation for subsequent electrochemical regulation. As illustrated in the battery assembly schematic, the AZO-modified separator is positioned between the NCA cathode and the lithium metal anode and immersed in liquid electrolyte, forming a sandwich-type cell configuration. In this design, the AZO layer directly faces the lithium anode, where its uniform ion/electron conduction characteristics are intended to guide lithium-ion flux and suppress localized dendrite growth.

**Figure 2:**
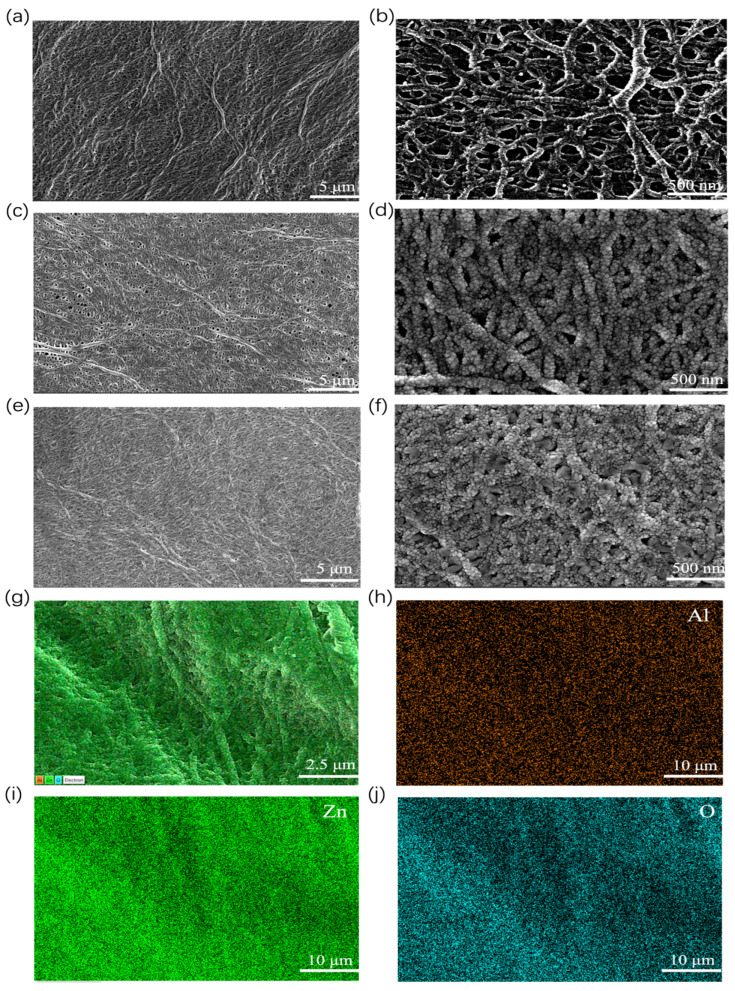
(**a**,**b**) SEM images of the pristine unmodified PE separator; (**c**,**d**) SEM images of the 150 nm AZO-modified film; (**e**,**f**) SEM images of the 200 nm AZO-modified film; (**g**–**j**) EDS elemental analysis of the AZO-modified film.

To establish a baseline for comparison and to assess the morphological changes induced by the AZO coating, the surface morphology of the pristine unmodified PE separator was first characterized by SEM. Figure 2a,b shows the surface morphology of the pristine PE separator at different magnifications. At low magnification (Figure 2a), the separator exhibits a highly developed three-dimensional interconnected porous network that is densely covered with micropores across a large macroscopic area. Upon increasing the magnification (Figure 2b), the intricate “spider web-like” interwoven characteristics of the micro-fibers and the detailed nanoscale pore structure become distinctly visible. with the pore sizes ranging from approximately 200 nm to 400 nm. This highly interconnected porous architecture not only ensures adequate electrolyte infiltration and wettability but also provides a physical barrier, to some extent, against the penetration of growing lithium dendrites.

Compared to the pristine separator, distinct morphological changes are observed after AZO deposition. As shown in Figure 2c,e, the AZO modification layers, deposited for different durations, achieve complete and uniform coverage over the microfibrillar skeleton of the PE separator. At low magnification, the modified separators maintain their original three-dimensionally interconnected net-like topological structure without significant pore blockage. High-resolution images (Figure 2d,f) further elucidate the growth evolution of AZO: the AZO deposits as fine nanoparticles that densely coat the surface of the PE microfibrils, resulting in a slight increase in fiber diameter. As a result of the coating deposition, the average pore size is slightly narrowed to approximately 100–200 nm for the 150 nm AZO-modified separator and further to 50–150 nm for the 200 nm AZO-modified version. With prolonged sputtering time, these nanoparticles further accumulate and coalesce on the fiber surfaces, forming a more continuous and compact coating. While preserving the highly interconnected porous network for rapid ion transport, the uniformly distributed lithiophilic AZO nanoparticles serve as effective heterogeneous nucleation sites. This can significantly reduce the lithium nucleation overpotential and guide the uniform deposition of lithium on the anode surface, thereby effectively suppressing dendrite growth. Elemental mapping (Figure 2g–j) further confirms the homogeneous distribution of Al, Zn, and O within the modification layer. This not only verifies that the magnetron sputtering process enables large-area uniform film deposition but also ensures consistent electrochemical activity across the separator surface. From a microstructural perspective, these findings elucidate the regulatory role of the AZO coating in modulating lithium deposition behavior and its underlying mechanism for enhancing the cycling stability of the battery.

**Figure 3:**
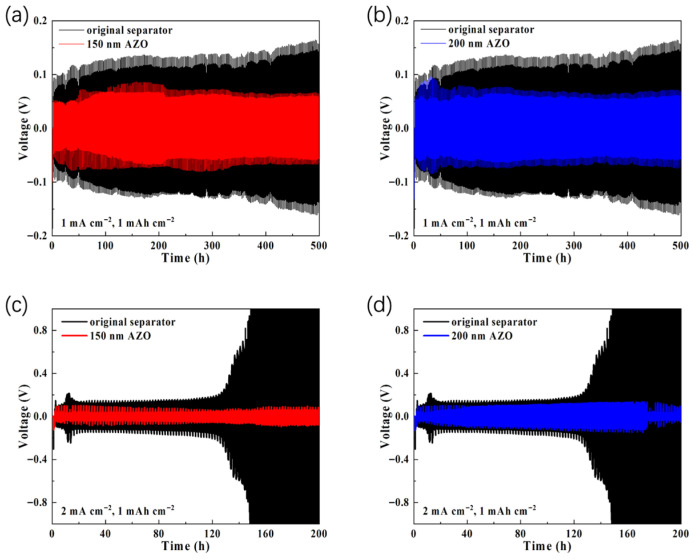
(**a**,**b**) Electrochemical performance of symmetric cells using 150 nm AZO-modified separator, 200 nm AZO-modified separator, and original blank separator at a current density of 1 mA cm^−2^ and areal capacity of 1 mAh cm^−2^; (**c**,**d**) Electrochemical performance of symmetric cells using 150 nm AZO-modified separator, 200 nm AZO-modified separator, and original blank separator at a current density of 2 mA cm^−2^ and areal capacity of 1 mAh cm^−2^.

The electrochemical performance of the AZO-modified separators was evaluated using Li||Li symmetric cells. As shown in Figure 3a,b, symmetric cells incorporating AZO-modified separators exhibit markedly lower and more stable overpotentials. Specifically, the initial overpotentials for cells with 150 nm and 200 nm AZO-modified separators are only 49 mV and 51 mV, respectively, accompanied by minimal voltage fluctuations. Compared to cells with the pristine separator, this superior performance is sustained with consistently low overpotentials throughout the subsequent 500 h of testing, demonstrating the stable electrochemical behavior of AZO-based cells.

To further assess the performance under more demanding conditions, the current density was increased to 2 mA cm^−2^ while maintaining a constant areal capacity of 1 mAh cm^−2^ (Figure 3c,d). Under these conditions, symmetric cells with AZO-modified separators continue to exhibit substantially lower overpotential than those with the pristine separator. At approximately 100 h, the overpotential of the cell employing the 150 nm AZO-modified separator is around 52 mV, and that of the cell with the 200 nm AZO-modified separator is approximately 72 mV—both significantly lower than the roughly 141 mV observed for the cell with the pristine separator. Specifically, the cell with the pristine separator began to exhibit signs of failure at approximately 130 h, accompanied by a marked increase in overpotential, while the cells employing AZO-modified separators maintained stable overpotentials over 200 h of cycling with minimal fluctuations. These results provide evidence for the effectiveness of AZO-modified separators in suppressing polarization in lithium metal symmetric cells under the tested conditions.

**Figure 4:**
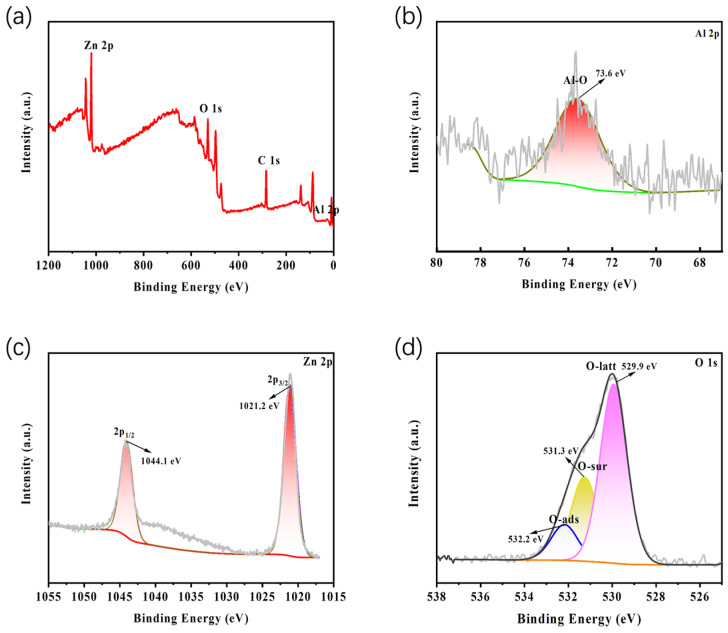
XPS analysis of the AZO film: (**a**) Survey spectrum; (**b**) Al 2p spectrum; (**c**) Zn 2p spectrum; (**d**) O 1s spectrum.

To further analyze the chemical composition of the AZO-modified films, XPS characterization was performed on the prepared AZO-modified films. As shown in Figure 4a, XPS survey spectrum analysis indicates the presence of Al, Zn, O, and C elements in the film. Figure 4b shows the high-resolution XPS spectrum of the Al 2p region, where the characteristic peak at 73.6 eV is attributed to Al 2p in the Al-O bond, confirming the substitution of Al^3+^ into the ZnO lattice [36]. In the Zn 2p spectrum (Figure 4c), the characteristic peaks at 1021.2 eV and 1044.1 eV correspond to Zn 2p_3/2_ and Zn 2p_1/2_ in the Zn-O bond, respectively, indicating that Zn primarily exists in the +2 oxidation state [36]. In the O 1s spectrum (Figure 4d), the broad peak can be deconvoluted into three components [37]: the characteristic peak at 529.9 eV corresponds to lattice oxygen (O^2−^) in the wurtzite AZO structure, the peak at 531.3 eV corresponds to defect oxygen (such as oxygen vacancies) on the sample surface, and the peak at 532.2 eV corresponds to chemisorbed oxygen or hydroxyl groups on the sample surface. These results comprehensively confirm, from a surface chemistry perspective, the successful preparation of an aluminum-doped zinc oxide (AZO) coating via magnetron sputtering. The prepared film is a composite system composed of Al, Zn, and O elements, where Al doping and the accompanying oxygen vacancy defects constitute the structural foundation for functional properties such as moderate conductivity.

To further investigate the role of the AZO coating in regulating initial lithium deposition, cross-sectional SEM images were acquired after the first charge in symmetric cells at 1 mA cm^−2^ and 1 mAh cm^−2^. As shown in Figures S1–S3 (Supplementary Materials), the morphology and thickness of the deposited lithium layer differ markedly between cells with pristine and AZO-modified separators. These observations provide direct visual evidence that the AZO coating effectively modulates lithium-ion flux and suppresses dendritic growth during the initial plating stage.

**Figure 5:**
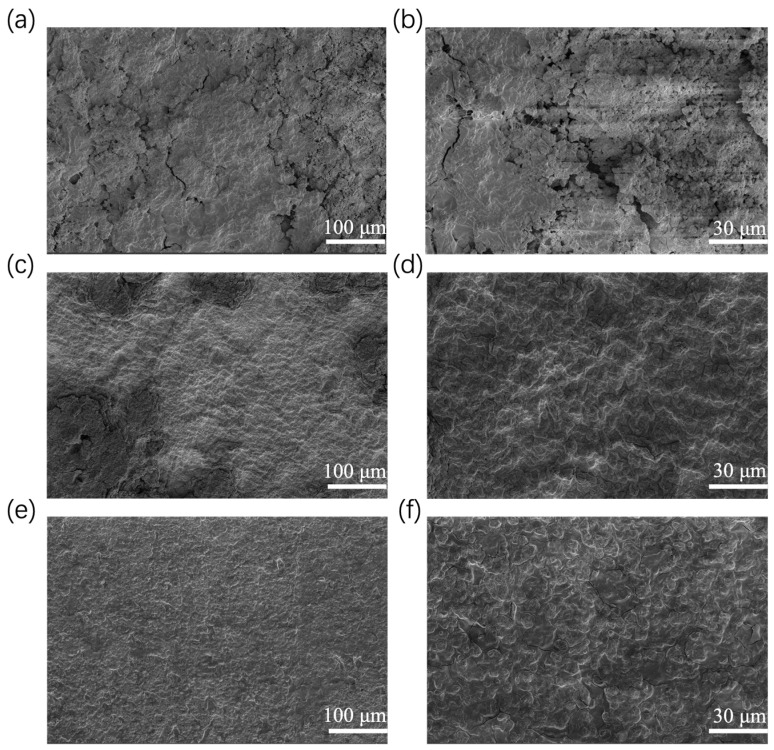
SEM morphological images of lithium deposition on electrode surfaces after 100 cycles in symmetric cells at 1 mA cm^−2^ current density and 1 mAh cm^−2^ areal capacity; (**a**,**b**) Lithium electrode corresponding to the blank separator; (**c**,**d**) Lithium electrode corresponding to the 150 nm AZO-modified separator; (**e**,**f**) Lithium electrode corresponding to the 200 nm AZO-modified separator.

Building on these findings from the initial deposition stage, the electrode surface morphology after 100 cycles was further examined to assess the long-term effectiveness of the AZO coating. As shown in Figure 5a, the lithium electrode cycled with the blank separator exhibits extensive, deep cracks (several micrometers wide) and a highly porous, mossy structure, indicating severe dendrite proliferation and extensive dead lithium formation. The higher-magnification image (Figure 5b) further reveals pronounced surface roughness with loosely stacked, filament-like deposits, confirming uncontrolled dendritic growth. In stark contrast, the electrode cycled with the 150 nm AZO-modified separator (Figure 5c,d) displays a remarkably smooth and compact surface, composed of densely packed, faceted block-like grains with no observable cracks or dendritic features, demonstrating effective suppression of dendrite growth and uniform lithium plating. For the 200 nm AZO-modified separator (Figure 5e,f), the surface remains entirely crack-free and densely covered with aggregated block-like deposits; however, occasional larger agglomerates are visible, suggesting a slightly less uniform deposition compared to the 150 nm coating. Nevertheless, both AZO-modified electrodes exhibit substantially denser and more homogeneous morphologies than the blank separator, providing direct visual evidence that AZO modification effectively mitigates dendrite growth and contributes to the enhanced cycling stability observed in the electrochemical tests.

To explore the performance of AZO-modified separators in lithium metal batteries with higher energy density, full cells assembled with high-loading NCA cathodes and lithium anodes were tested at a rate of 0.33 C.

**Figure 6:**
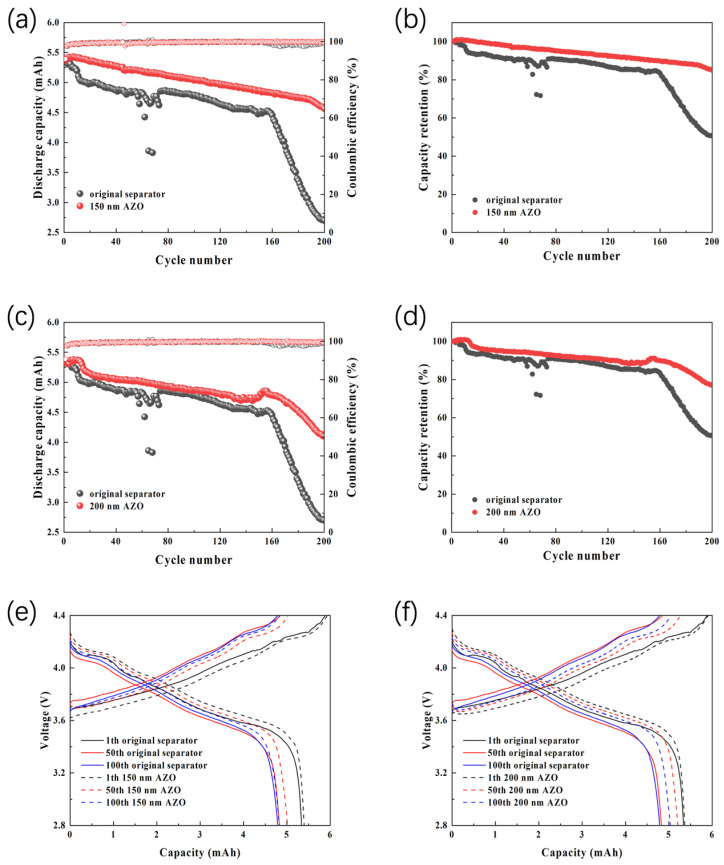
Electrochemical performance of Li||NCA full cells with AZO-modified separators and the original blank separator. (**a**,**b**) Cycling performance and capacity retention of Li||NCA with 150 nm AZO-modified separator and original blank separator at 0.33 C rate; (**c**,**d**) Cycling performance and capacity retention of Li||NCA with 200 nm AZO-modified separator and original blank separator at 0.33 C rate; (**e**) Charge–discharge voltage profiles at different cycles for Li||NCA with 150 nm AZO-modified separator and original blank separator; (**f**) Charge–discharge voltage profiles at different cycles for Li||NCA with 200 nm AZO-modified separator and original blank separator.

Figure 6a–d presents the discharge capacities and corresponding capacity retention ratios of full cells assembled with 150 nm and 200 nm AZO-modified separators. The initial discharge capacities of these cells are 5.37 mAh and 5.32 mAh, respectively. After 80 and 200 cycles, the cell employing the 150 nm AZO-modified separator retains capacities of 5.11 mAh and 4.56 mAh, corresponding to retention rates of 95.1% and 84.9%. Although the cell with the 200 nm AZO-modified separator exhibits slightly lower overall performance than its 150 nm counterpart, it still delivers relatively impressive capacities of 4.93 mAh and 4.10 mAh at the same cycle numbers, with retention rates of 92.6% and 77.1%, respectively. In contrast, the control cell using the pristine separator shows markedly lower retention rates of only 89.9% and 50.5% after 80 and 200 cycles. These results suggest that AZO modification can enhances both the discharge capacity and cycling stability of Li||NCA full cells. The charge–discharge voltage profiles in Figure 6e,f further corroborate this improvement. At equivalent operating voltages, cells equipped with AZO-modified separators consistently deliver higher capacities than the control cell. Throughout the entire cycling test, the AZO-modified cells maintain higher average capacities and superior capacity retention, providing strong support for the effectiveness of the AZO coating strategy.

To gain deeper insight into the interfacial kinetics and the origin of the enhanced cycling stability, electrochemical impedance spectroscopy (EIS) was performed on full cells assembled with the 150 nm AZO-modified separator and the pristine PE separator. The evolution of charge transfer resistance during cycling was analyzed, and the detailed results are presented in Figure S4a,b (Supplementary Materials). These measurements provide additional evidence for the improved interfacial stability afforded by the AZO coating.

The superior electrochemical performance of AZO-modified separators can be attributed to the synergistic interplay between their multifunctional composite architecture and the precisely controllable fabrication process. First, the moderate electrical conductivity imparted by aluminum doping enables uniform electron transport within the AZO layer, promoting a homogeneous current distribution at the electrode interface and effectively reducing local overpotentials. This fundamentally suppresses the initiation and growth of lithium dendrites. Second, the uniformly distributed AZO nanoparticles on the separator surface exhibit excellent lithiophilic properties, serving as efficient heterogeneous nucleation sites [38,39,40] that direct uniform lithium-ion deposition and facilitate the formation of a dense and smooth lithium metal morphology. Third, while preserving the intrinsic three-dimensional porous network of the pristine separator, the dense and homogeneous AZO coating itself functions as a robust physical barrier that inhibits dendrite penetration, while still allowing adequate electrolyte infiltration and rapid lithium-ion transport. Finally, the magnetron sputtering process enables precise control over film thickness, nanoscale compositional uniformity, and strong interfacial adhesion between the AZO layer and the separator substrate. This ensures sustained structural integrity of the coating throughout prolonged cycling and provides a stable interfacial environment for the lithium plating/stripping process, thereby substantially enhancing the cycling stability and capacity retention of full cells.

## 4. Conclusions

In this study, a uniform AZO modification layer was successfully deposited onto PE separators by magnetron sputtering and subsequently applied in lithium metal batteries.

The AZO-modified separator effectively combines the structural robustness of conventional ceramic coatings with moderate interfacial conductivity. By enabling homogeneous current distribution, reducing local overpotentials, and providing lithiophilic nucleation sites, the AZO layer synergistically promotes uniform lithium-ion deposition and substantially suppresses dendrite growth. In symmetric cell tests, separators modified with 150 nm and 200 nm AZO coatings exhibited low initial overpotentials of 49 mV and 51 mV, respectively, at a current density of 1 mA cm^−2^, and maintained stable overpotentials over 500 h. At an increased current density of 2 mA cm^−2^, the overpotentials of cells using 150 nm and 200 nm AZO-modified separators were approximately 52 mV and 72 mV at 100 h, respectively—considerably lower than that of the cell with the pristine separator (141 mV). Moreover, post-cycle electrode surfaces exhibited dense, uniform lithium deposition with no observable dendrites. In full cells paired with NCA cathodes, the AZO-modified separator delivered a high capacity retention of 84.9% after 200 cycles at 0.33 C, significantly surpassing the 50.5% retention of the control cell with an unmodified separator. These results demonstrate that constructing a multifunctional AZO coating via magnetron sputtering represents a feasible and scalable separator modification strategy, offering an effective technical route toward the development of lithium metal batteries with both high energy density and enhanced safety.

## Data Availability

The original contributions presented in this study are included in the article. Further inquiries can be directed to the corresponding authors.

## Supplementary Materials

The following supporting information can be downloaded at: https://www.mdpi.com/article/10.3390/ma19071429/s1.
