# Supplementary material for: High-Stability Lithium Metal Batteries Enabled by AZO-Modified Separators
Source: Materials (Basel). 2026 Apr 3;19(7):1429. doi: 10.3390/ma19071429 (PMC13074370; doi:10.3390/ma19071429)
Supplement: Supplementary file 1 [file materials-19-01429-s001.zip › materials-4206074-supplementary.pdf]

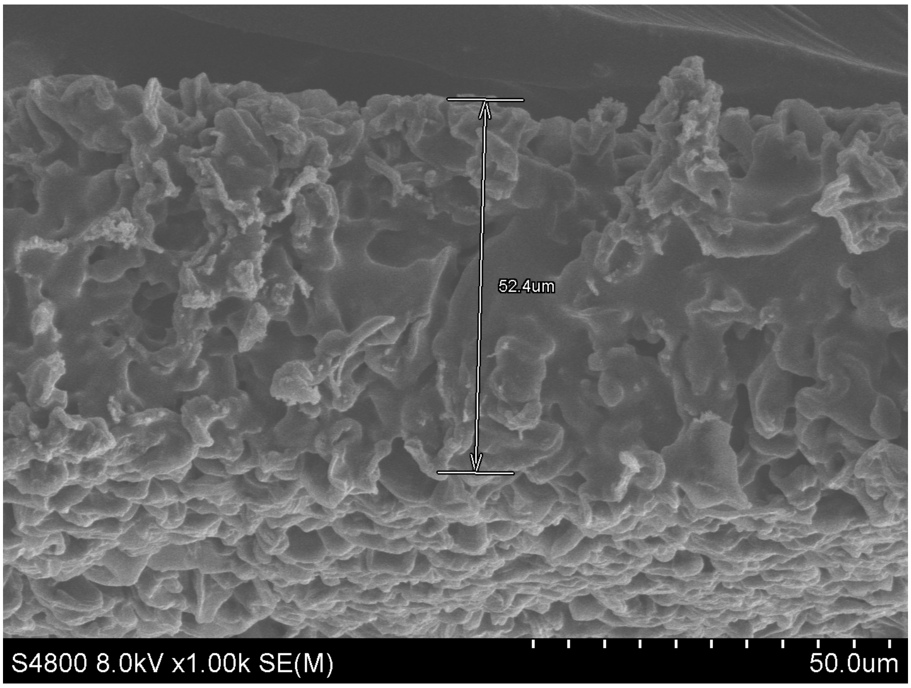

Figure S1. Cross-sectional SEM image of lithium deposition on the electrode surface after the first charge in a symmetric cell assembled with the pristine separator at a current density of 1 mA cm^{-2} and an areal capacity of 1 mAh cm^{-2}.

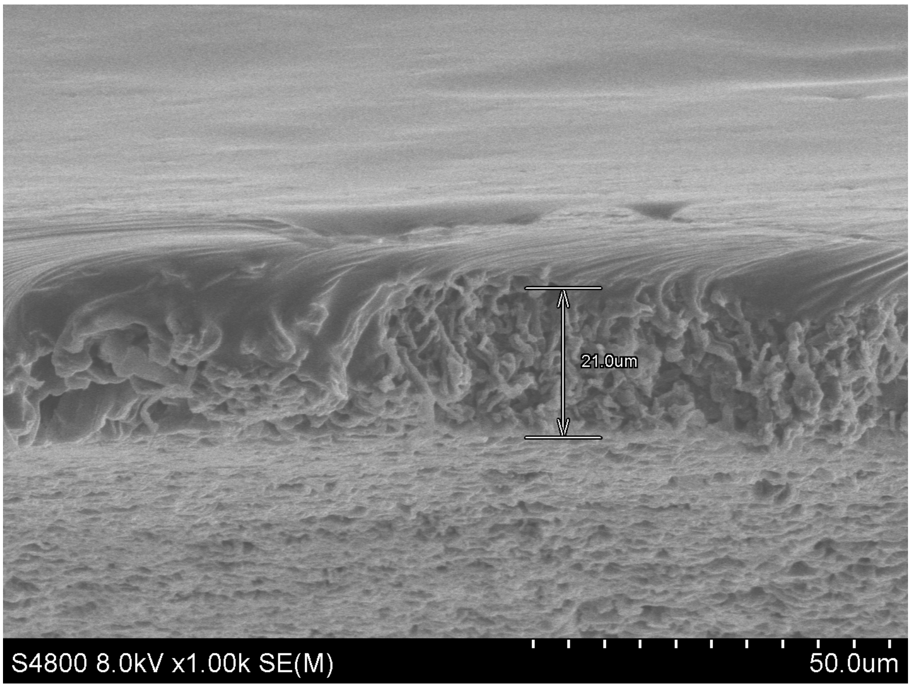

Figure S2. Cross-sectional SEM image of lithium deposition on the electrode surface after the first charge in a symmetric cell assembled with the 150 nm AZO-modified separator at a current density of 1

mA cm^{-2} and an areal capacity of 1 mAh cm^{-2}.

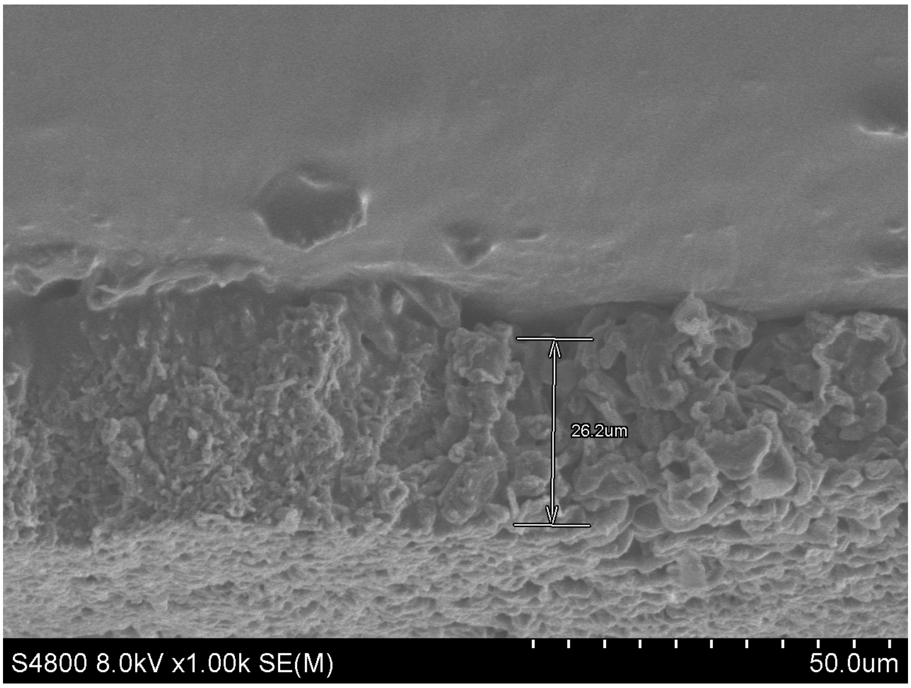

Figure S3. Cross-sectional SEM image of lithium deposition on the electrode surface after the first charge in a symmetric cell assembled with the 200 nm AZO-modified separator at a current density of 1 mA cm^{-2} and an areal capacity of 1 mAh cm^{-2}.

To provide direct evidence of the effect of AZO modification on lithium dendrite suppression, cross-sectional SEM images were acquired from symmetric cells after the first charging step at a current density of 1 mA cm^{-2} and an areal capacity of 1 mAh cm^{-2}.

Figure S1 shows the lithium electrode cycled with the pristine PE separator. The deposited lithium layer exhibits a loose, porous, and irregular dendritic morphology, with a thickness of approximately 52.4 μm. Such a thick and disordered structure indicates the occurrence of interfacial side reactions and uncontrolled dendrite growth during the initial plating.

In contrast, the introduction of the AZO-modified separator leads to an improved lithium deposition morphology. As shown in Figure S2 (150 nm AZO) and Figure S3 (200 nm AZO), the thickness of the deposited lithium layer is reduced to 21.0 μm and 26.2 μm, respectively. More importantly, the lithium deposits become compact and uniformly flat, with few mossy or needle-like loose dendrites observable. This evolution from a porous, dendritic structure to a dense, smooth layer provides visual evidence that the AZO coating effectively homogenizes the lithium-ion flux and suppresses disordered dendrite growth during electrochemical deposition.

Figure S1. Cross-sectional SEM image of lithium deposition after the first charge in a symmetric cell using the pristine PE separator. The deposited layer thickness is approximately 52.4 μm, showing a loose and dendritic morphology.

Figure S2. Cross-sectional SEM image of lithium deposition after the first charge in a symmetric cell using the 150 nm AZO-modified separator. The deposited layer thickness is approximately 21.0 μm, exhibiting a compact and uniform structure.

Figure S3. Cross-sectional SEM image of lithium deposition after the first charge in a symmetric cell using the 200 nm AZO-modified separator. The deposited layer thickness is approximately 26.2 μm, also displaying a dense and smooth morphology.

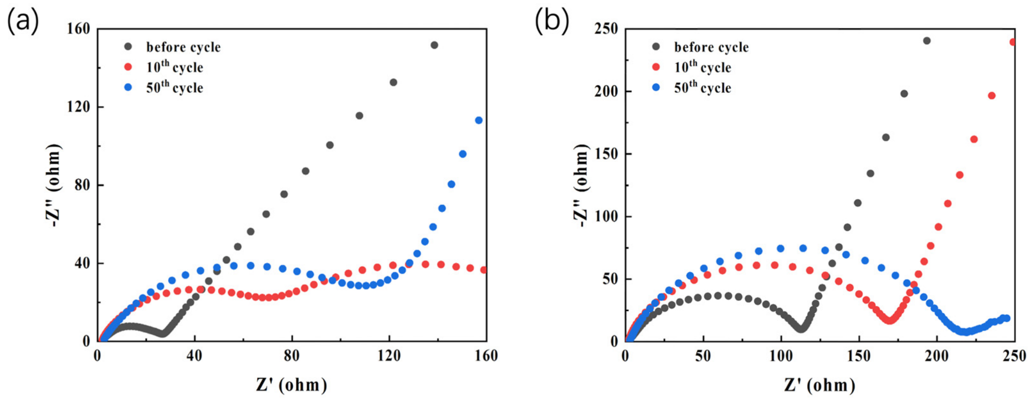

Figure S4. (a) Nyquist plots of the 150 nm AZO-modified separator after different cycle numbers at a rate of 0.33 C; (b) Nyquist plots of the bare separator after different cycle numbers at a rate of 0.33 C.

To investigate the evolution of charge transfer resistance during battery cycling, EIS was performed on full cells assembled with the 150 nm AZO-modified separator and the pristine PE separator. The measurements were conducted after different numbers of cycles at 0.33 C. The interfacial charge transfer resistance (Rct) was estimated from the semicircle observed in the high-frequency region of the Nyquist plots.

Figure S4a shows the Nyquist plots of the cell with the 150 nm AZO-modified separator. The initial Rct is relatively low (approximately 30 Ω). After 10 and 50 cycles, the Rct values increase to 67 Ω and 110 Ω, respectively, indicating a gradual but moderate increase in interfacial resistance over cycling.

Figure S4b presents the Nyquist plots of the cell with the pristine PE separator. The initial Rct is higher than that of the AZO-modified cell. More importantly, after 10 and 50 cycles, the Rct values increase to 170 Ω and 220 Ω, respectively—substantially higher than

those observed for the AZO-modified cell at the same cycle numbers.

These results demonstrate that the AZO-modified separator maintains a lower and more stable charge transfer resistance during cycling compared to the pristine separator, confirming the improved interfacial stability provided by the AZO coating.
